# Modeling the effects of neuronal morphology on dendritic chloride diffusion and GABAergic inhibition

**DOI:** 10.1186/1471-2202-15-S1-P138

**Published:** 2014-07-21

**Authors:** Namrata Mohapatra, Fidel Santamaria, Peter Jedlicka

**Affiliations:** 1 Institute of Clinical Neuroanatomy, Neuroscience Center, Goethe-University, Frankfurt, Germany; 2 Biology Department and Neurosciences Institute,The University of Texas at San Antonio, San Antonio, USA

## 

Gamma-aminobutyric acid receptors (GABA_A_Rs) are ligand-gated chloride (Cl^−^) channels which mediate the majority of inhibitory neurotransmission in the CNS. Spatiotemporal changes of intracellular Cl^−^ concentration alter the concentration gradient for Cl^−^ across the neuronal membrane and thus affect the current flow through GABA_A_Rs and the efficacy of GABAergic inhibition. However, the impact of complex neuronal morphology on Cl^−^ diffusion and the redistribution of intracellular Cl^−^ is not well understood. Recently, computational models for Cl^−^ diffusion and GABA_A_R-mediated inhibition in realistic neuronal morphologies became available [1-3]. Here we have used computational models of morphologically complex dendrites to test the effects of spines on Cl^−^ diffusion. In all dendritic morphologies tested, spines slowed down longitudinal Cl^−^ diffusion along dendrites and decreased the amount and spatial spread of synaptically evoked Cl^−^ changes. Spine densities of 2-10 spines/µm decreased the longitudinal diffusion coefficient of Cl^−^ to 80-30% of its value in smooth dendrites, respectively. These results suggest that spines are able to limit short-term ionic plasticity [4] at dendritic GABAergic synapses.
